# Clinical Insights into MicroRNAs in Depression: Bridging Molecular Discoveries and Therapeutic Potential

**DOI:** 10.3390/ijms25052866

**Published:** 2024-03-01

**Authors:** Lalit Kaurani

**Affiliations:** Department for Epigenetics and Systems Medicine in Neurodegenerative Diseases, German Center for Neurodegenerative Diseases (DZNE), 37075 Göttingen, Germany; lalit.kaurani@dzne.de

**Keywords:** microRNA, depression, major depressive disorder, biomarker, antidepressant, miRNA therapeutics, extracellular vesicles

## Abstract

Depression is a major contributor to the overall global burden of disease. The discovery of biomarkers for diagnosis or prediction of treatment responses and as therapeutic agents is a current priority. Previous studies have demonstrated the importance of short RNA molecules in the etiology of depression. The most extensively researched of these are microRNAs, a major component of cellular gene regulation and function. MicroRNAs function in a temporal and tissue-specific manner to regulate and modify the post-transcriptional expression of target mRNAs. They can also be shuttled as cargo of extracellular vesicles between the brain and the blood, thus informing about relevant mechanisms in the CNS through the periphery. In fact, studies have already shown that microRNAs identified peripherally are dysregulated in the pathological phenotypes seen in depression. Our article aims to review the existing evidence on microRNA dysregulation in depression and to summarize and evaluate the growing body of evidence for the use of microRNAs as a target for diagnostics and RNA-based therapies.

## 1. Introduction

### 1.1. General Introduction to Depression

Depression, an overarching term encompassing a spectrum of mood disturbances, stands as a predominant global health concern, currently recognized as a principal contributor to disability across the world. An estimated 5% of the global adult population is impacted by depression, a number witnessing an alarming ascent each year [1,2,3]. Cumulative lifetime prevalence statistics, when evaluated on an international scale, suggest that about one in seven individuals will grapple with a depressive episode at some juncture in their lives, with rates ranging between 11.1% and 14.6% [4]. Notably, while depressive episodes can manifest at various life stages, spanning early adolescence to adulthood, the median onset age gravitates around the mid-20s [3,5]. Gender disparities are also evident; women exhibit nearly double the susceptibility to experiencing depression during their lifetimes compared to their male counterparts, though this ratio demonstrates considerable cross-national variation [6,7].

The etiology of depression is multifaceted, interwoven with both environmental contributors such as socioeconomic conditions and biological determinants, including genetic predispositions [3,8]. The interplay between genes and the environment is further modulated by epigenetic mechanisms, adding another layer to its complexity [9]. Among the various forms of depression, major depressive disorder (MDD) is particularly well-researched, although its pathophysiology is yet to be fully deciphered. Available evidence points towards molecular disruptions, including neurotransmitter imbalances and augmented inflammatory cytokine levels, underscoring their intricate molecular foundation [10]. This comprehensive review focuses on the clinical evidence and provides a nuanced understanding of miRNAs in the context of depression, offering insights into their diagnostic and therapeutic potential in both MDD and the wider spectrum of depressive disorders.

### 1.2. Current Therapeutic Approaches

The main goal of treatment for depression is an accurate diagnosis and the remission of symptoms. Depression is clinically identified as ‘major depression’ when it meets the symptomatic criteria outlined in the Diagnostic and Statistical Manual of Mental Disorders, Fourth Edition, 2000 (DSM-V) [11]. Diagnostic measures are based on clinical observation, although no objective biological markers currently exist. The main approaches to treatment can be separated into four broad categories: generic psychosocial interventions, formulation-based interventions of psychological therapy, pharmacotherapy, and electroconvulsive therapy (ECT) [8]. In mild cases of depression, psychosocial and psychological intervention may be sufficient, while in more severe cases, pharmacotherapy is also required. Most pharmacological interventions for depression enhance the transmission of monoamines through different routes, with the most common first-line treatments being selective serotonin reuptake inhibitors and serotonin–norepinephrine reuptake inhibitors. Despite the range of psychological and pharmacological therapies available, many patients do not achieve remission [12,13].

### 1.3. General Introduction to miRNAs

MicroRNAs are small non-coding RNAs of approximately 19–22 nucleotides that function to inhibit the level of target genes within discrete regulatory networks [14]. Over 60% of human protein-coding genes contain conserved miRNA target sites [15]. The interaction networks of miRNAs are complex and vast; one miRNA can target many mRNAs, and one mRNA can be regulated by many different miRNAs [16,17]. Their identification in many different organisms also suggests their regulatory mechanisms are evolutionarily conserved [18]. According to recent work by the Functional Annotation of the Mammalian Genome (FANTOM5) consortium, approximately half of miRNAs show cell type specificity, and one-quarter are more broadly expressed [19].

In the central nervous system, miRNAs play a role in neurodevelopment, synaptic plasticity, and stress responses, thus providing a molecular link to psychiatric conditions [20,21,22]. Several lines of evidence have already shown their involvement in other neurological and mental health disorders [22]. 

In fact, miRNAs are differentially expressed in depression, as identified by a multitude of recent studies, pointing to their involvement here as well [23]. miRNAs have emerged as key players in depression due to their role as post-transcriptional regulators of gene expression and their ability to pass the blood–brain barrier [24]. Excitingly, they offer new avenues for understanding the intricate molecular underpinnings of depression. Their capacity to modulate multiple gene networks associated with neuroplasticity and stress response, as well as their shuttling between the blood and the brain, makes miRNAs compelling targets for both diagnostic and therapeutic interventions in depressive disorders.

## 2. Role of miRNAs in Neurobiology

### 2.1. Biogenesis of miRNAs

**Figure 1:**
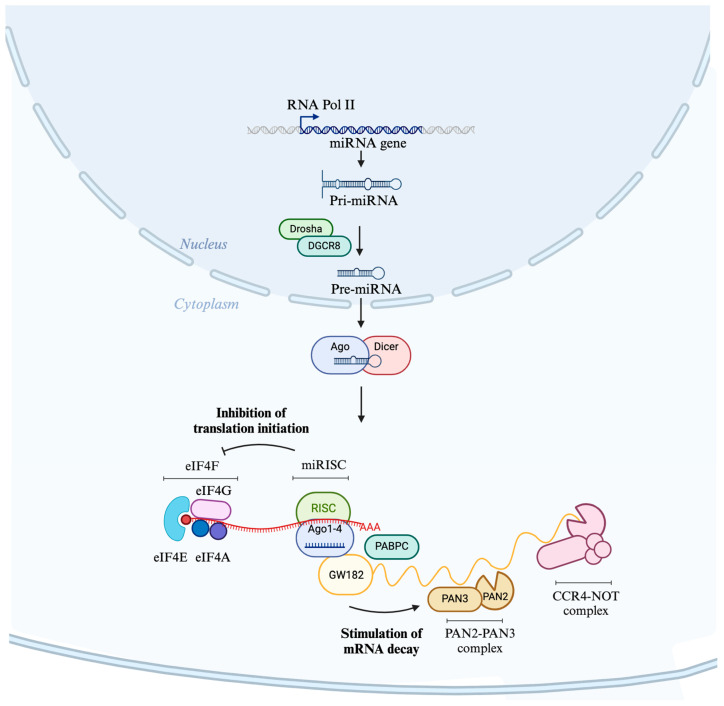
Biogenesis and mechanisms of miRNA-mediated gene regulation. This figure illustrates the miRNA gene expression pathway and subsequent miRNA-mediated gene silencing mechanisms. The process begins in the nucleus, where the miRNA gene is transcribed by RNA polymerase II into a primary miRNA (pri-miRNA). The pri-miRNA is then processed by the Drosha-DGCR8 complex into a precursor miRNA (pre-miRNA), which is exported to the cytoplasm. In the cytoplasm, the pre-miRNA is further cleaved by the Dicer enzyme, with the assistance of the Argonaute (Ago) proteins, to form a mature miRNA. This miRNA is incorporated into the RNA-induced silencing complex (miRISC), which includes Ago1-4 proteins. The miRISC complex can inhibit translation initiation through its interaction with eukaryotic initiation factors (eIFs), particularly eIF4F and eIF4A, or stimulate mRNA decay by recruiting the GW182 protein, which in turn associates with the PAN2-PAN3 and CCR4-NOT deadenylation complexes, leading to the shortening of the poly(A) tail and degradation of the target mRNA. Created with BioRender.com. (Retrieved from https://app.biorender.com/biorender-templates, accessed on 30 January 2024).

Biogenesis of miRNAs begins when genomic regions encoding miRNAs are transcribed by RNA polymerase II into primary miRNAs [25]. miRNAs are transcribed largely from intergenic regions, but many are also found within intronic regions [26]. These primary transcripts are first processed by the microprocessor complex consisting of the ribonuclease III enzyme Drosha and the RNA-binding protein DGCR8 to yield a ~70 nucleotide pre-miRNA [27]. The pre-miRNA is then exported to the cytoplasm by exportin-5. Exportin-5 recognizes the 3′ overhang of the pre-miRNA hairpin, binding to it and facilitating its transport through the nuclear pore complex, and it is there processed by Dicer to yield a mature miRNA duplex (Figure 1). Within this duplex, one miRNA is from the 3′ and one is from the 5′ strand of the precursor; however, one is often a ‘guide’ strand, predominant in amount and biological activity [28].

### 2.2. Mode of Action of miRNAs

miRNAs function as part of the miRNA-induced silencing complex (miRISC), composed of a ‘guide’ strand of the final duplex loaded onto an Argonaute (AGO) protein. The complex binds usually within the 3′ untranslated region of target RNAs through base pairing at the seed sequence, with the strongest interactions being with sites that complement miRNA nucleotides 2–8 [28,29]. miRISC binding to its target results in gene silencing by inhibiting translation at the initiation step and mediating mRNA decay (Figure 1) [16,17,30,31]. The inhibition of translation initiation is caused by interfering with eIF4A-I and eIF4A-II functions by inducing their dissociation from mRNAs and consequent inhibition of assembly of the eIF4F translation initiation complex [32,33]. mRNA decay involves the recruitment of a member of the glycine-tryptophan protein of the 182 kDa (GW182) protein family, which interacts with polyadenylate-binding protein (PABPC), promoting mRNA deadenylation through PAN2–PAN3 and CCR4–NOT complexes and consequent degradation by exonucleases [34,35]. Although miRNAs function intracellularly as part of the miRISC to mediate gene silencing, their influence is not restricted to the intracellular milieu. Their ability to be secreted and circulated systemically expands their potential roles and implications, particularly in the nervous system. This brings us to their extracellular journey, a phenomenon essential for understanding their utility as diagnostic markers and therapeutic agents.

### 2.3. Circulation of miRNAs

Emerging evidence underscores that miRNAs are not confined to the cellular environment but can be actively released into extracellular fluids, where they circulate systemically to modulate distant target cells [36]. A portion of these circulating miRNAs associate with proteins such as AGO2, which exists in a free-floating form. However, their primary extracellular transport mechanism involves exosomes, a specialized subtype of extracellular vesicles (EVs).

EVs are lipid bilayer-enclosed particles secreted by a plethora of cell types, encapsulating diverse cargo, including but not limited to nucleic acids such as mRNA and miRNA [37,38,39]. These vesicles are categorized chiefly into ectosomes and exosomes based on their cellular origin: ectosomes are formed by the outward budding of the plasma membrane, while exosomes result from the fusion of multivesicular endosomes with the plasma membrane [38,40].

The intricacies of vesicle biogenesis and cargo loading are dictated by a complex interplay involving the Endosomal Sorting Complex Required for Transport (ESCRT), syntenin-Alix pathway, tetraspanins, various lipids, and the arrestin domain-containing protein 1 [41,42,43,44,45]. Once secreted, EVs navigate through the extracellular matrix and vascular barriers, eventually merging into the circulatory and integumentary systems [40]. In the realm of neuroscience, exosomes are of unique significance for their nanoscale dimensions (30–200 nm), which enable them to cross the blood-brain barrier [37]. While the exact mechanisms of this transport are still under investigation, transcytosis appears to be a plausible route [46]. Upon reaching the target site, the vesicle’s cargo is internalized by the recipient cell through endocytosis and subsequently released into the cytosol via fusion with the endosomal or lysosomal membrane, often in an acidification-dependent manner [47].

### 2.4. Role of miRNAs in Neural Function: From Synaptic Architecture to Plasticity

#### 2.4.1. Synaptic Localization and Functional Roles of miRNAs in Neuronal Cells

miRNAs have been identified as vital regulatory entities across numerous physiological systems, with their role being especially profound in the nervous system. A significant majority of miRNAs, approximately 70%, are expressed in the brain, where they are implicated in multiple neural functions such as neurogenesis and dendritic spine formation [48,49].

The synaptic network of the brain is marked by complex dendritic interconnections and far-reaching axons, posing a challenge for the swift and effective transport of molecules to remote synaptic sites. Neurons have adapted to this by synthesizing proteins locally at synapse sites, enabling precise regulatory control. Studies have revealed the presence of miRNAs, mRNAs, and polyribosomes within various neural components such as dendrites, axons, and dendritic spines [50]. Notably, certain miRNAs are more concentrated in synaptic fractions compared to whole-cell lysates, suggesting specialized roles at these sites. This was further substantiated by Lugli G et al., who, through subcellular fractionation methods, discovered a rich presence of miRNAs in synaptoneurosomes, in pre- and post-synaptic densities of dendritic spines. These studies also highlighted a specific group of miRNAs enriched in synaptic fractions in contrast to entire cell lysates [50,51].

Regarding the origin of mature miRNAs in neuronal segments, it is either via local conversion of pre-miRNAs or through transport from the cell body. The presence of miRNA precursors and the Dicer enzyme, crucial for RNA processing, has been noted in dendritic and axonal areas [50,52,53,54]. The co-existence of pre-miRNAs and Dicer in these areas implies a mechanism that ensures miRNA specificity and rapid conversion to mature forms as required [55,56]. Emerging research further hints that synapses may actively participate in transforming precursor miRNAs into mature forms, possibly influenced by NMDA receptor activity [52,57,58]. A hypothesis that synaptic activation might be instrumental in this conversion process has been proposed [59,60], though it requires further exploration. The mechanisms governing the delivery and localization of miRNAs to distal synapses are a subject of ongoing research. Specific binding mechanisms, such as the interaction between DEAH-box helicase DHX36 and pre-miR-134, aid in the transport of miRNAs [52]. 

Functionally, miRNAs play nuanced roles at the synapse. Certain miRNAs, such as miR-132, miR-134, and miR-9-3p, have been identified as key players in normal synaptic functioning and neuropathologies associated with synaptic plasticity, including Alzheimer’s disease [61,62,63]. Their functional roles are also deeply intertwined with activity-dependent translation processes. Examples include the intricate interactions of miR-138 with *Lypla1* mRNA or the competition between the RNA-binding protein HuD and miR-129 [64,65]. The interplay of miRNAs, mRNAs, and their associated proteins shapes synaptic functions, such as the onset of long-term synaptic depression (LTD) [66,67]. These findings underscore the nuanced role of miRNAs in synaptic dynamics and neurological health.

#### 2.4.2. Regulatory Influence of miRNAs on Synaptic Transmission and Neural Plasticity

miRNAs exert influence on synaptic structure by modulating the components of the post and pre-synaptic terminal. Specific studies highlight the role of miRNAs such as miR-125b, miR-223, miR-137, and miR-146a-5p in regulating postsynaptic glutamate receptors. For instance, hippocampal neuron dynamics reveal that miR-125b manages the GluN2A component of the NMDA receptor [68], while miR-223 exerts control over the GluN2B NMDA receptor subunit and the AMPA receptor’s GluA2 subunit [69]. Furthermore, miR-137 has associations with the AMPA receptor, particularly its GluA1 subunit [70], and intriguingly, miR-146a-5p, while not directly linked to glutamate receptors, influences them via its interaction with the microtubule-associated protein 1B [71]. Not limited to postsynaptic interactions, miRNAs also target presynaptic components and signaling proteins. As an example, miR-188’s association with the semaphorin-3F receptor Nrp-2 impacts the abundance of dendritic spines in hippocampal neurons [72]. Meanwhile, synaptic glutamate release in Drosophila is influenced by miR-1000’s regulation of the vesicular glutamate transporter [73]. Additionally, miR-137’s reach extends beyond the AMPA receptor, affecting proteins vital for synaptic vesicle discharge [70].

On a broader scale of synaptic plasticity dependent on protein synthesis, miRNAs, including miR-26a and miR-384-5p, have shown alterations in concentration in response to synaptic triggers. Their interactions during NMDA receptor-dependent LTP have consequences for the ribosomal S6 kinase 3 (RSK3) translational regulator [74]. In various synaptic plasticity forms, miR-124 and miR-22 act through distinct targets, with miR-124, for example, influencing NMDA receptor-dependent LTP via Zif268 [75] and miR-137 engaging with mGluR-dependent LTD in hippocampal processes [70].

## 3. MicroRNAs and Depression

### 3.1. miRNAs in the Pathophysiology of Depression

Emerging research underscores noticeable shifts in miRNA levels in both the brain and periphery of individuals with depression. Generally, the predominant research paradigms encompass the following: (a) a comparative study of miRNA profiles between patients and controls; (b) an exploration of miRNA modulations subsequent to antidepressant interventions. In the former approach, the prefrontal cortex emerges as a primary brain region of interest. In the periphery, researchers often rely on whole blood, plasma, or serum samples. The second methodology is predominantly engaged to discern miRNA shifts following therapeutic regimens. A less conventional, albeit more invasive, medium is cerebrospinal fluid to probe miRNA dysregulation. Downstream analysis usually involves bioinformatics-driven approaches to identify miRNA–mRNA interactions, facilitating an understanding of the broader regulatory networks at play. Furthermore, to decode the functional dynamics of miRNA regulation, in vitro evaluations and animal model studies are occasionally integrated, though they are less prevalent. 

Some of the first studies to identify the role of microRNAs in the pathophysiology of MDD have examined the postmortem human brain. In one example study, the levels of miR-182 expression were disrupted in dentate gyrus granule cells of individuals with depression [76,77]. In the anterior cingulate cortex, a brain region critical for emotional and mood regulation, a depression-specific miRNA expression profile was identified compared to controls [78]. In a similar approach, miR-1202 was found to be downregulated in the prefrontal cortex of individuals with depression compared to controls [79]. This microRNA was found to be enriched in the brain ad negatively correlated with *GRM4* expression, a metabotropic glutamate receptor. Importantly, this study also showed that in individuals who had attained remission, levels of miR-1202 were increased.

While the postmortem brain remains a key system for investigating the molecular mechanisms of MDD, a drawback remains that microRNAome profiling in brain tissue at the time of death or sample collection may not be reflective of the disease course [22]. In recent years, miRNAs have been assessed in the periphery of individuals with depression. An advantage of this approach is capturing real-time miRNA differences between patient and control populations and quantifying miRNA changes in patients in response to treatment. One of the first studies to take this approach showed decreased miR-135a levels in patients with depression compared to controls, which had increased after 3 months of selective serotonin reuptake inhibitor treatment or cognitive behavioral therapy [80]. Another study by Belzeaux and colleagues identified a distinct set of miRNAs that are dysregulated in patients with depression, which could predict clinical improvement and treatment response [81]. A further study showed that with electroconvulsive therapy for treatment-resistant depression, levels of miR-223-3p could predict treatment response [82]. 

Despite numerous studies, there remains a substantial lack of consensus on key miRNAs and their functions in depression. To address this, recent advancements in the field have developed methods for the isolation of cell-type-specific microRNAs in the periphery. For example, microRNAs originating in different brain cell types can now be selected. Pioneering work taking this approach has examined miRNAs within neuron-derived extracellular vesicles in the periphery and has found decreased miR-93 levels in individuals with depression [83]. In another study comparing the neuron-derived extracellular vesicle microRNAome before and after treatment with escitalopram, a signature of miR-21-5p, miR-30d-5p, and miR-486-5p was found to be associated with treatment response [84]. 

An aggregated list of studies characterizing miRNA variations in depression highlighted in this review can be found in Table 1.

### 3.2. miRNAs as Diagnostic Biomarkers

The search for non-invasive peripheral biomarkers that mirror changes in the brain is a high priority in research on depression. Such biomarkers could substantially impact current clinical diagnosis practices and, if capable of predicting antidepressant response, the assignment of efficacious treatments. In this regard, circulating miRNAs in blood and other bodily fluids have emerged as promising candidates. Owing to their remarkable stability in circulation and the detectability of brain-specific miRNAs such as miR-9 and miR-223-3p [82,88], miRNAs offer a new avenue in biomarker research. 

#### 3.2.1. Blood-Derived miRNAs: Refining the Complex Landscape of Depression Biomarkers

Differential expression of miRNAs in the periphery has been consistently identified in depression and following treatment with antidepressants. Thus, it is clear that the etiology and therapeutic intervention in depression rely at least in part on regulation by miRNAs. 

One seminal study highlighted the plasticity of miRNA profiles following treatment, showing that a twelve-week therapeutic regimen of escitalopram promoted the upregulation of 28 microRNAs while concomitantly downregulating miR-34c-5p and miR-770-5p in the blood of individuals with depression [87,89] (see Table 2). In another study, an eight-week citalopram treatment normalized blood concentrations of two deregulated miRNAs, miRNA-132 and miR-124 [90]. While a four-week intervention with citalopram normalized the levels of 10 previously deregulated miRNAs [91]. A particularly noteworthy observation common to these therapeutic interventions was the consistent upregulation of miR-335 [89,91], previously shown to regulate glutamate metabotropic receptor 4 (*GRM4*), implicated with anxiety-associated behaviors [91]. Another study observed that blood levels of miR-1202 decreased in individuals with depression, a trend that was rectified following an eight-week treatment course of either escitalopram or desvenlafaxine [79,92]. Notably, both miR-1202 and miR-335 have been implicated in regulating GRM4 signaling pathways, thereby influencing stress-related behaviors [79]. Further adding to the complexity, a different study indicated that treatment responses to escitalopram, desvenlafaxine, and duloxetine were associated with changes in miR-1202 and miR-16-5p levels [93]. 

In parallel to the analysis of discrete microRNAs, several studies have opted for a more comprehensive approach employing small RNA sequencing (sRNA-seq). Using this approach, Belzeaux et al. showed that miR-3688 and miR-5695 served as predictive markers for exacerbated suicidal ideation in individuals subjected to an eight-week course of duloxetine or a placebo [94]. Similarly, Yrondi et al. employed next-generation sequencing (NGS) and observed that pharmacological interventions with escitalopram affected levels of 45 microRNAs, with miR-185-5p being particularly correlated with the reduced incidence of nausea, a common side effect associated with selective serotonin reuptake inhibitors (SSRIs) [95]. In another study, Lopez et al. utilized small RNA sequencing to investigate miRNAs as potential biomarkers for antidepressant responses in MDD within a randomized placebo-controlled trial of duloxetine. The research, conducted before and 8 weeks post-treatment, identified that miR-146a-5p, miR-146b-5p, miR-425-3p, and miR-24-3p exhibited differential expression linked to treatment outcomes. Furthermore, these miRNAs were shown to regulate the MAPK/Wnt signaling pathways, suggesting their significant role in the antidepressant response [86].

In sum, these studies substantiate the proposed use of circulating miRNAs in blood as biomarkers for depressive disorders and treatment responses. Moreover, the findings highlight the complex molecular interplay underlying depression and pharmacological interventions, setting the stage for future research that can enable more targeted and efficacious therapeutic strategies.

#### 3.2.2. Fluid-Based miRNA Landscapes: Serum, Plasma, and Cerebrospinal Fluid

The body of evidence linking specific miRNA profiles to depression extends beyond blood to encompass serum, plasma, and cerebrospinal fluid (CSF). For instance, two studies reported elevated serum and plasma levels of miR-182 and miR-132 in MDD, with both miRNAs found to modulate BDNF expression in neuronal cells [77,90]. Key studies show that inhibiting miR-182-5p alleviates depression-like behaviors in mouse models, suggesting its potential as a therapeutic target [98]. The upregulation of miR-182 reduces BDNF in the hippocampus, further underlining its role in depression pathophysiology [85]. A separate investigation by Wang et al. revealed that diminished plasma levels of miR-144-5p were correlated with depressive symptoms and reversed following eight weeks of personalized antidepressant treatment [96]. Rodent models also imply a role for miR-144-5p in hippocampal functioning, specifically through the regulation of the PTP1B protein, a known modulator of BDNF/TrkB signaling [99]. Several studies reported that many miRNAs, including miR-1202, miR-135a-5p, miR-184, let-7g-5p, miR-103a-3p, miR-107, miR-16-5p, miR-34a-5p, miR-451a, miR-221-3p, and let-7d-3p, found in CSF and serum, were dysregulated in depression and responded to antidepressant treatments [97,100,101,102,103].

#### 3.2.3. Extracellular Vesicles: Emerging Protagonists in the Biomarker Landscape of Depression

In the exploration of biomarkers for depression, extracellular vesicles (EVs) have recently come under investigation [104]. As previously described, EVs serve as a robust reservoir of miRNAs, facilitating their circulation between the brain and peripheral blood, and vice versa. EVs have been implicated in inflammatory disorders [105,106], cancer [107], and neurodegenerative diseases [108], with their role in psychiatric conditions gaining increasing interest [109]. For example, high-throughput miRNA sequencing identified differentially expressed miRNAs in the serum exosomes of patients with depressive symptoms [24]. Upregulated miRNAs were linked to the modulation of neurotrophic signaling pathways, while downregulated ones were associated with the regulation of apoptosis and immune functions [24]. Notably, the levels of miR-139-5p in serum exosomes distinguished individuals with MDD from healthy controls and could also induce depressive-like behaviors in murine models [110].

**Figure 2:**
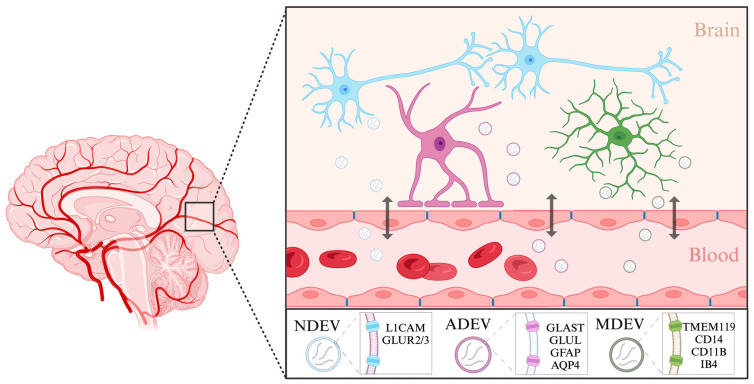
Characterization of brain-derived extracellular vesicles. Brain-derived extracellular vesicles enable cell-to-cell communication between different cell types in the brain as well as in the periphery. Extracellular vesicles can originate in neurons (NDEVs), astrocytes (ADEVs), and microglia (MDEVs). These vesicles can shuttle across the blood–brain barrier and enter the body’s circulation. Listed are the markers specific to each cell type that have been used to isolate extracellular vesicles from solution. Created with BioRender.com. (Retrieved from https://app.biorender.com/biorender-templates, accessed on 30 January 2024).

Of particular interest is the potential for isolating brain-derived extracellular vesicles (BDEVs) from peripheral blood, which offers a window into central nervous system-specific molecular landscapes [111,112]. BDEVs can originate from many different cell types in the brain, including neurons, astrocytes, and microglia (Figure 2). Neuron-derived EVs have been isolated previously using a biotinylated antibody to L1 cell adhesion molecule (L1CAM), a transmembrane protein specific to neurons [113]. They could also be labeled using antibodies against the GluR2/3 subunits of AMPA receptors [114]. Using this approach, studies have already begun to assess the roles of neuron-derived EVs in depression. Elevated levels of miR-17-5p in neuron-derived EVs correlated with subthreshold depressive symptoms [112]. Moreover, neuronal EVs isolated from individuals with depression undergoing 8-week escitalopram treatment displayed brain-specific proteins and miRNAs (such as miR-30d-5p and miR-486-5p) and also exhibited size alterations reversible by the antidepressant therapy. Changes in miRNA cargo in these neuron-derived EVs also reflected varying responses to antidepressant interventions [84].

The isolation of astrocyte and microglia-derived EVs is also starting to be explored. One of the main forms of communication between neurons and astrocytes is through EVs, where they can have both neuroprotective and pathological properties, highlighting their importance [115]. Astrocyte-derived EVs (ADEVs) have previously been isolated from human blood using a biotinylated antibody to glutamine aspartate transporter (GLAST), an astrocyte-specific membrane protein, glutamine synthetase (GLUL) [116], as well as using glial fibrillary acidic protein (GFAP) and aquaporin 4 (AQP4) [117]. Using this approach, ADEVs have been implicated in modulating immune responses during stress and depression [118,119]. It has been shown that individuals with depression have larger numbers of GFAP and AQP4/GFAP-positive EVs in the blood than healthy controls, suggesting increased leakage of astrocyte-derived EVs through the blood–brain barrier. In a recent study, the miRNA content of ADEVs from individuals with depression was found to have significantly deregulated miR-9 levels, suggesting a specific involvement of this miRNA in the disease’s pathophysiology [120].

Similar to astrocytes, microglia also rely on EVs for cell-to-cell communication within the brain. Largely, the isolation of microglia-derived EVs from complex fluids such as blood samples has so far also been conducted using antibodies or lectins against microglial surface markers [121]. The heterogenous nature of microglial cells is inevitably reflected in their surface protein content and cargo and should thus be used to inform isolation protocols. For instance, in a stroke experimental model, EVs from microglia were isolated using markers characteristic of their specific activation state, namely TMEM119 and CD14 [122]. In another example, small EVs of microglial origin were isolated using CD11B, a more general marker of microglia [123]. Microglia or macrophage-derived EVs have also been isolated using myeloid marker IB4 [124]. Examining the contents of cell-type specific EVs enables a more targeted understanding of disease mechanisms. Further efforts to characterize extracellular vesicles and their components which originate in different brain cell types will improve our understanding of disease mechanisms.

## 4. Therapeutic Potentials of miRNAs in Depression

miRNAs have emerged as promising molecular targets in the quest to develop innovative interventions for depression. By understanding their intricate regulatory roles, novel therapeutic strategies harnessing the power of miRNAs may revolutionize the treatment landscape for this debilitating condition.

### 4.1. Current Therapeutic Strategies

Recent studies highlight the therapeutic potential of miRNA-based treatments in a range of conditions, including depression [125,126,127,128]. Although the majority of this research remains in the preclinical phase and, to the best of our understanding, there have been no clinical trials targeting depression using miRNA-based therapies. It is evident that substantial endeavors are still required to make headway in this domain.

miRNA therapeutic strategies primarily modulate the levels of target miRNAs, either by amplifying or attenuating their levels, using oligonucleotides to emulate or curtail miRNA activity, or utilizing small molecules to modulate miRNA functionality [129]. These compounds are introduced into cells using methods such as viral vectors that encode miRNA mimics or antagonists, nanoparticle carriers including liposomes, or cellular vesicles such as exosomes and microvesicles [130,131,132]. Effective in vivo delivery of miRNAs is crucial, aiming to prevent RNase-mediated degradation, enhance targeting precision, and minimize unwanted immune responses. Historically, intravenous and localized approaches have been the primary delivery techniques for in vivo miRNA applications. However, recent investigations are also considering oral and intranasal methods for administration [130,131].

#### 4.1.1. miRNA Inhibition Therapy

**Figure 3:**
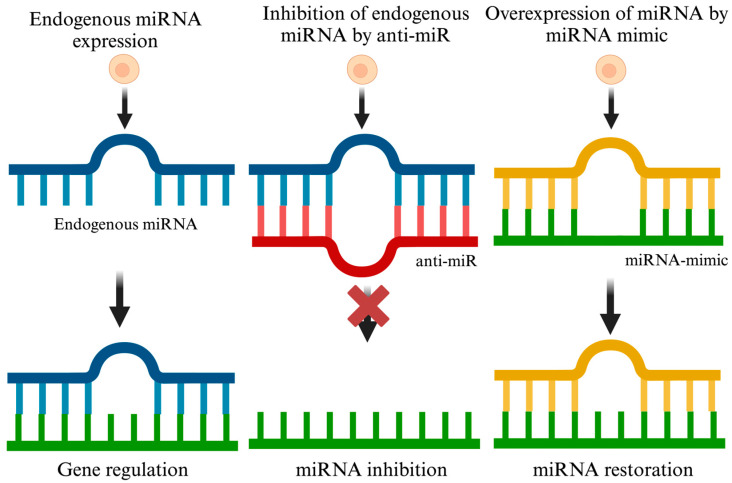
Modulation of miRNA function: endogenous expression, inhibition, and restoration strategies. This figure delineates three key approaches to modulating miRNA activity. On the left, ‘Endogenous miRNA expression’ illustrates a native miRNA being transcribed and subsequently leading to gene regulation through interaction with target mRNA. The middle panel, titled ‘Inhibition of endogenous miRNA by anti-miR’, depicts the process of an anti-miR molecule binding to and inhibiting an endogenous miRNA, thereby preventing it from exerting its gene regulatory function. The rightmost panel, ‘Overexpression of miRNA by miRNA mimic’, shows a miRNA mimic being introduced to overexpress a specific miRNA, leading to gene regulation. This schematic representation highlights the potential of manipulating miRNA levels and activity, either by inhibiting their function to upregulate gene expression or by mimicking their activity to downregulate gene expression in various therapeutic contexts. Created with BioRender.com.

The purpose of miRNA inhibition therapy is to counterbalance miRNAs, which have a heightened presence in depressive conditions. The goal is to recalibrate the expression and operations of genes to their natural state. Various methods, including miRNA inhibitors such as antisense miRNA oligonucleotides, locked nucleic acid (LNA) antisense, miRNA antagomirs, and miRNA sponges, have been explored to reduce miRNA expression and counter upregulated miRNAs [133,134,135]. In this review, we refer to all these approaches collectively as anti-miR, which are designed to decrease target miRNA expression. Anti-miRs typically comprise 17–22 nucleotides and are tailored to be complementary to the target miRNA, effectively reducing their expression by binding to them [134]. When anti-miRs bind to their respective target miRNAs, they form double-stranded molecules with the miRNA of interest, preventing these miRNAs from interacting with the Ago complex and their target mRNA. This interruption halts the translational inhibition caused by dysregulated miRNAs in disease conditions (Figure 3).

Anti-miRs that include LNA are engineered for enhanced stability and stronger binding affinity to their target miRNAs. Furthermore, studies have shown that LNA-containing anti-miRs exhibit increased solubility in water and culture media, making them more suitable for in vivo applications [136,137,138]. For example, the research by Islam et al. demonstrated that LNA-based anti-miRs (targeting miR-181a-5p, miR-146a-3p, and miR-148a-5p) effectively silenced these overexpressed miRNAs in the CA region in mice, leading to improved cognition and reduced inflammation [139]. Apart from LNAs, miRNA sponges are also utilized to inhibit upregulated miRNAs. These RNA constructs, equipped with multiple artificial miRNA binding sites, function by competing with cellular miRNAs for their targets [140,141].

Recent advancements in animal-based research have been instrumental in identifying novel targets for mitigating the symptoms of depression. A promising area of study involves the modulation of Notch 1, aimed at improving neural connectivity. Inhibition using miR-9 has been explored as a potential therapeutic strategy [142,143,144]. Furthermore, investigations using chronic unpredictable mild stress (CUMS) have revealed its role in triggering major depression through the downregulation of excitatory synapses, particularly affecting their presynaptic targets on GABAergic neurons in the nucleus accumbens [145]. This disruption in GABAergic activity leads to an imbalance between excitatory and inhibitory signals in the brain, undermining the integrity of neural circuits. Such imbalances are key contributors to the onset of MDD and other stress-related conditions [146]. Notably, research has shown that inhibiting miR-15b-5p using anti-miR in the nucleus accumbens can substantially decrease the occurrence of CUMS-induced depression and restore the reduced levels of excitatory synapses [145].

Another key brain region involved in depression is the hippocampus. Under the regulatory control of miR-18a-5p is hippocampal serotonergic balance, and anti-miRs may be a strategic move to promote resiliency to depression through glucocorticoid signaling [147]. Another explored target is miR-132, which is a class of miRNAs that works in the neuro-immune compartment and has been linked to elevated BNDF levels in in vivo settings [148,149]. Similarly, elevated miR-202-3p levels in the hippocampus have been linked to diminished *BDNF* expression in a CUMS-induced depression model. The inhibition of miR-202-3p in the hippocampus has demonstrated an antidepressant-like effect, significantly reducing depressive behaviors in the CUMS model, highlighting the therapeutic potential of miRNA modulation in depression treatment strategies [150].

Strategies such as miR-134-5p inhibition have also yielded positive neuronal outcomes and reduced depression-like behaviors in specific models [151]. A noteworthy development in the field is the identification of red blood cell-specific miR-144-3p as a potential biomarker. This biomarker not only assists in the diagnosis of depression but also predicts the response to ketamine treatment in stress-susceptible mice and MDD patients. Intriguingly, the use of an anti-miR to miR-144-3p, administered subcutaneously, has led to a marked reduction in depression-related phenotypes in stress-prone mice [152]. Similarly, the increased expression of miR-96 has led to a decrease in the synaptic vesicle glycoprotein 2 family protein which is associated with depression-like behavior, while inhibiting miR-96 using anti-miR decreased depression-like behavior and memory impairment in mice [153].

#### 4.1.2. miRNA Restoration Therapy

Within the sphere of depression research, ‘miRNA mimics’ are designed as a double-stranded RNA with a guide strand that reflects the sequence of the associated endogenous miRNA. Using these small RNA mimics, the levels of select miRNAs can be transiently overexpressed, helping researchers understand the consequent biological effects. Focusing on miRNA restoration therapy, this approach aims to reverse miRNA deficiencies associated with depressive disorders. It involves using synthetic miRNA mimics or employing viral vectors that express pertinent miRNAs (Figure 3). These mimics, serving as substitutes for natural miRNAs, are incorporated into the miRISC, leading to alterations in the activity of downstream mRNA targets [154]. The success of this miRNA restoration method has been validated through a range of cell-based and in vivo experiments. Additionally, the field of miRNA restoration is exploring the use of miRNA expression vectors, including adenoviral, lentiviral, and retroviral vectors, as another promising avenue.

A key study by Kota et al. provides insight into this strategy, demonstrating an example where the levels of miR-26 were significantly lower under specific conditions, in contrast to their normal abundance [155]. Reintroducing miR-26 into these cells slowed their cell cycle progression. Remarkably, treating cells with a recombinant adenovirus carrying miR-26 resulted in enhanced cell recovery and a decrease in cell proliferation, without any adverse effects observed.

In the context of depression, notable miRNAs which may have therapeutic implications or serve as viable targets include let-7, miR-144, miR-9, miR-16, miR-30a, miR-133b, miR-26a-2, and several others. Recent studies have focused on the potential therapeutic modulation by the let-7 family, examining the effects of physical activity on its epigenetic regulation [156]. Advances in lentiviral technologies have been harnessed to overexpress let-7d, showing promise for anxiolytic and antidepressant outcomes [157]. Another noteworthy study observed a decrease in miR-139-5p levels in CUMS-induced mice, correlating with depression-like symptoms. The increasing level of miR-139-5p expression in the hippocampus through AAV technology resulted in the activation of the cAMP/PKA signaling pathway, demonstrating antidepressant-like effects in these mice [158].

A similar methodology showed that increasing the levels of miR-144 lead to observed antidepressant effects in the CUMS rat model [99]. It is essential to recognize the potential of miR-144, which is typically reduced in MDD patients [159]. Animal models have confirmed that levels of miR-144 are upregulated in response to treatment with mood stabilizers [160]. Complementing these findings is the interesting case of miR-124-3p in depression. Studies have shown a notable upregulation of miR-124-3p in the hippocampus of CUMS mice. This upregulation plays a crucial role in reducing proinflammatory cytokines and in the deactivation of microglia [161]. Highlighting its significance, recent research by Ge et al. identified the presence of miR-124-3p within microglial EV miRNA cargos. This presence is linked to the alleviation of neurodegenerative processes and the improvement of cognitive functions, marking miR-124-3p as a potential therapeutic intervention in depression [162].

Strikingly, certain miRNAs exhibit inherent antidepressant properties in various animal models, making them also an appealing target for testing as miRNA mimic therapies. Examples include miR-26a-2, which acts on serotonergic neurons [125,163], and miR-101, which reverses specific depressive behaviors [164]. Notably, combining miR-135a with traditional antidepressants resulted in decreased inflammatory markers in certain models [80].

### 4.2. Future Directions

miRNAs have emerged as a key player in the complex molecular mechanisms underpinning depression. Their ability to regulate a myriad of genes post-transcriptionally places them in a unique position to be therapeutic targets. As we progress into an era emphasizing precision medicine, the potential to measure and manipulate miRNAs in minimally invasive ways offers promising avenues for treating depression. This section explores the emerging targets, innovative drug delivery methods, and potential improvements to current strategies.

#### 4.2.1. Emerging Targets in the miRNA Landscape

**Figure 4:**
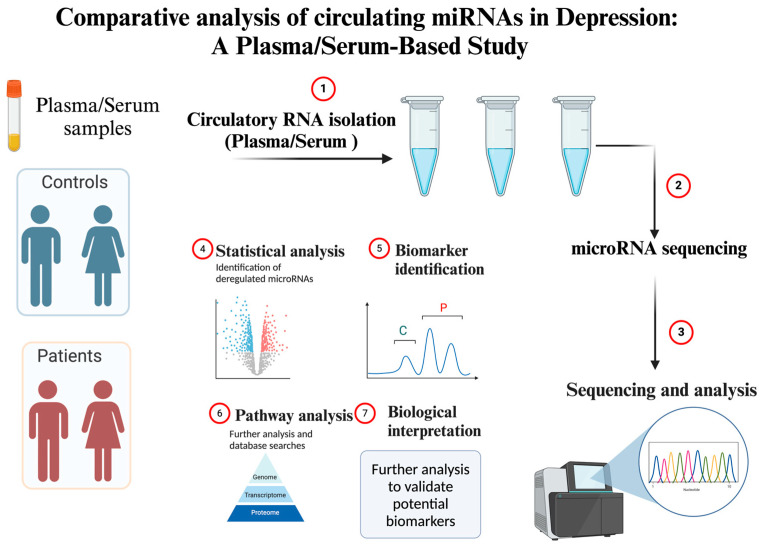
Methodological framework for miRNA biomarker discovery in depression. This figure delineates a structured approach for the identification and analysis of circulating miRNAs as potential biomarkers in the context of MDD. The process begins with the collection of plasma/serum samples from both patients diagnosed with MDD and healthy control subjects. Step 1 involves the isolation of circulatory RNA from these samples, which is then subjected to microRNA sequencing (Step 2), a critical phase where the miRNAs are identified and quantified. In Step 3, the sequencing data undergo rigorous analysis. Step 4 involves statistical analysis to detect miRNAs whose expression levels significantly differ between the two groups. Biomarker identification (Step 5) is achieved through the comparison of miRNA expression profiles, leading to the selection of candidate miRNAs for further validation. Pathway analysis (Step 6) is then conducted to understand the biological pathways affected by the dysregulated miRNAs, followed by a detailed biological interpretation (Step 7) to elucidate their potential roles in the pathophysiology of MDD. This comprehensive protocol aims to enhance the understanding of miRNA functions in MDD and support the development of new therapeutic strategies. C = Control/healthy individuals; P = Patients. Created with BioRender.com.

As miRNA research gains momentum, novel targets are constantly being identified. Recent studies have highlighted miRNAs such as miR-16, miR-135a, miR-144, miR-26a-2, and miR-1202 as potential biomarkers in MDD [165]. Their differential expression levels in individuals with depression have been consistently reported. Moreover, their roles have been intricately linked with serotonin pathways, neuroinflammation, and neurogenesis, key pathways in brain functioning. As more miRNAs are identified, it is imperative to validate their potential therapeutic roles, understand their mechanistic pathways, and explore their interactions with other miRNAs or mRNA targets. Integrative omics approaches, coupling miRNA profiling, transcriptomics, proteomics, and network analysis, will be essential in this endeavor. Figure 4 in the manuscript visually encapsulates the methodology of a study design aimed at analyzing these miRNA candidates in plasma or serum samples from individuals with MDD compared to healthy controls, thereby providing a foundation for the biological interpretation of their roles in the disease pathology.

#### 4.2.2. Drug Delivery—Crossing the Biobarriers

The therapeutic application of miRNAs is hindered by the challenges posed by their stability, specificity, and delivery. However, recent advances promise to address these hurdles. miRNA delivery is categorized into the following methods. 

Viral-based and non-viral-based: Viral vectors, such as adenoviruses (Ads), have historically been used in gene therapy. First-generation Ads faced challenges due to their immunogenic nature, but subsequent iterations proved more promising. Ad-based miRNA delivery has been notably successful in mitigating cardiomyocyte hypertrophy in mice [166,167]. In contrast, adeno-associated viruses (AAVs) are recognized for being non-pathogenic and exhibiting reduced immunogenicity. Notably, AAV9 vectors demonstrated the capability to cross the blood–brain barrier, promising potential in treating central nervous system diseases [168]. Numerous studies have employed AAVs to modulate miRNA levels in vivo. A compelling example of this is the research conducted by Huang et al., where AAV technology was used to elevate miR-139-5p levels in the hippocampus of mice [158]. On the other hand, Herpes simplex viruses (HSVs) possess a natural inclination for nerve cells, and their application in delivering miR-124 resulted in the modification of neuron-specific alternative splicing [169]. Nevertheless, concerns about the vector’s size and possible cytotoxicity remain.

On the flip side, non-viral vectors offer a range of options. A type of lipid-based vectors, liposomes are spherical vesicles encapsulating an aqueous core. The cationic nature of some liposomes facilitates the binding of negatively charged miRNAs, safeguarding them from degradation [170,171,172]. Complexes of liposomes and nucleic acids, termed lipoplexes, have been employed to transport miRNAs into cells [130,173]. Inorganic nanoparticles such as gold nanoparticles are also under exploration for miRNA delivery, given their biocompatibility and modifiability [174,175]. Silica-based nanoparticles have also been employed to co-deliver anti-miR-221, although this approach has mostly only been used in the cancer field [176,177].

Exosomes: These naturally occurring vesicles can be engineered to carry miRNAs, ensuring effective delivery across the blood–brain barrier (BBB). They possess several features that make them particularly attractive for the delivery of biomolecules, including miRNAs: natural biocompatibility, low immunogenicity, and the inherent ability to cross biological barriers such as the BBB [178]. Exosomes can encapsulate and protect miRNA molecules from degradation in the extracellular environment, ensuring their safe passage to target cells. Once they reach their destination, exosomes can release their miRNA cargo into different cell types in the brain, modulating gene levels and potentially ameliorating molecular pathways contributing to depressive symptoms [179,180].

Several studies have demonstrated the feasibility and efficacy of exosome-mediated miRNA delivery in neurological conditions. For example, exosomes loaded with miRNA-124, known for its neuroprotective properties, facilitated recovery from ischemic conditions in a mouse model [181]. Such successes hint at the potential of a similar approach in depression therapy. Additionally, the source of exosomes can play a role in enhancing therapeutic outcomes. Exosomes derived from mesenchymal stem cells (MSCs), for instance, have shown promise due to their anti-inflammatory properties [182,183], which could be particularly beneficial in conditions such as depression, where neuroinflammation is a known contributor.

However, while the potential is immense, there are challenges. Optimizing exosome loading with desired miRNAs, ensuring target specificity, and scaling up production for clinical applications are areas that need focused research. Moreover, understanding the long-term effects of exosome-mediated miRNA delivery and ensuring minimal off-target effects is crucial.

Peptide-based delivery: Peptide-based delivery systems have emerged as a promising approach for the targeted delivery of therapeutic molecules, including miRNAs, to specific cells or tissues. This stems from the versatility, biocompatibility, and low toxicity of peptides, which can be designed to interact specifically with cell surface receptors or to traverse cell membranes efficiently [184,185]. In the context of depression, the central nervous system (CNS) remains a challenging target due to the restrictive nature of the BBB. The effective delivery of miRNA molecules to the brain is imperative for therapeutic efficacy. Peptides, with their ability to be tailored for specificity, offer a potential solution.

Certain peptides, such as those derived from rabies virus glycoprotein (RVG), have shown promise in specific targeting of neuronal cells [186,187]. By conjugating these peptides with miRNAs, one can achieve efficient transport across the BBB and subsequent delivery to neuronal cells. Such delivery ensures that therapeutic miRNAs reach their targets, maximizing therapeutic potential while minimizing off-target effects.

Another advantage of peptide-based delivery is the potential for co-delivery of multiple therapeutic agents. Peptides can be designed to encapsulate or bind to both miRNAs and other therapeutic molecules, allowing for a synergistic approach to treatment. This is especially relevant in conditions such as depression, where a multifactorial etiology might necessitate a combination of therapeutic interventions. Moreover, the ease of synthesis and modification of peptides allows for rapid optimization of delivery vectors. This adaptability means that peptide-based delivery systems can be quickly refined based on in vitro and in vivo feedback, accelerating the translational potential of miRNA therapies for depression.

However, as with all therapeutic approaches, challenges persist. Ensuring the stability of the peptide-miRNA complex in systemic circulation, avoiding rapid degradation, and preventing immune responses are areas that warrant attention. Additionally, understanding the long-term effects of repeated administration and potential peptide-related toxicity is essential for clinical translation.

#### 4.2.3. Augmenting Existing Strategies

While the therapeutic potential of miRNAs in depression is evident, the following can help refine current strategies.

Combination Therapies: Co-administering miRNA modulators with conventional antidepressants can potentiate therapeutic effects and mitigate potential side effects. Illustratively, in one experiment, miR-135a was combined with standard antidepressant treatments, leading to a notable decrease in inflammatory markers in a mouse model exhibiting depression-like behaviors.

Bioinformatics and Personalized Medicine: Leveraging bioinformatics tools and machine learning algorithms can help predict miRNA-disease associations and individual miRNA profiles. This facilitates a personalized medicine approach, where treatments are tailored based on an individual’s unique genetic makeup.

#### 4.2.4. Lateral Flow Assay for miRNA Detection

**Figure 5:**
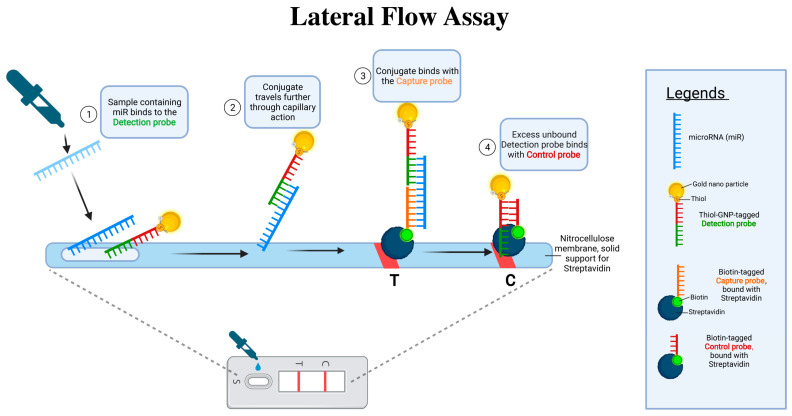
miRNA detection using a gold nanoparticle-based lateral flow assay. This figure presents the sequential steps of a lateral flow assay (LFA) for the detection of microRNA (miRNA). Step 1: A sample (blood, plasma/serum) containing miRNA is applied to the assay, where the miRNA binds to a thiol-GNP-tagged detection probe. Step 2: The conjugate of miRNA and detection probe migrates along the nitrocellulose membrane through capillary action. Step 3: Upon reaching the test line (T), the conjugate encounters a biotin-tagged capture probe bound to streptavidin, facilitating binding and indicating a positive result. Step 4: Any excess detection probe that does not bind to the miRNA continues to migrate to the control line (C), where it is captured by a biotin-tagged control probe bound to streptavidin, confirming the assay’s functionality. The presence of two distinct lines indicates a successful assay, with the test line corresponding to miRNA detection and the control line serving as a procedural control. The legends section defines the visual symbols used in the figure, such as the miRNA, gold nanoparticle, thiol, thiol-GNP-tagged detection probe, biotin, streptavidin, and the biotin-tagged capture and control probes. Created with BioRender.com.

One of the challenges in harnessing miRNAs as therapeutic targets is their accurate and rapid detection. The lateral flow assay (LFA), similar to the mechanism of a home pregnancy test, provides a quick, user-friendly, and cost-effective platform to detect specific biomolecules, including miRNAs. By using tailored probes that can bind to miRNAs associated with depression, LFA can serve as a diagnostic tool to determine miRNA deregulation in blood samples [188,189]. Such a tool is invaluable, not just for initial diagnosis but also for monitoring the efficacy of any miRNA-based therapy, allowing for dynamic treatment strategies tailored to individual patient needs (see Figure 5).

The key advantages of using LFA for detecting miRNAs in depression include speed, simplicity, and the potential for point-of-care testing. It does not require sophisticated laboratory equipment, making it feasible for routine clinical settings or even potential home use. However, there are limitations. The sensitivity and specificity of the LFA need to be rigorously validated against gold-standard miRNA detection methods. Additionally, while changes in specific blood miRNA levels may correlate with depressive states, the biological underpinnings of these changes and their direct causal relationship to depression remain to be fully elucidated.

## 5. Challenges and Limitations

The therapeutic potential of miRNAs in depression is a promising yet challenging frontier in molecular psychiatry. Advancements in miRNA profiling have not yet overcome the difficulty of capturing the full spectrum of miRNA interactions and their complex regulatory networks. This limitation is compounded by inconsistencies in results across similar studies, likely due to variations in sample processing, sequencing methodologies, and bioinformatic analyses. Advocating for standardized procedures is critical for achieving reliable outcomes.

Another significant hurdle is the efficient delivery of miRNA-based therapeutics across the blood–brain barrier. Despite the emergence of innovative methods such as nanoparticles and viral vectors, issues with delivery specificity and efficiency remain unresolved. Moreover, the broad regulatory roles of miRNAs raise concerns about off-target effects and unintended physiological changes, which could worsen depressive symptoms or introduce new clinical problems. This risk is exacerbated by the challenge of targeting specific miRNAs without inadvertently influencing other pathways, given the promiscuous nature of miRNA–mRNA interactions.

Finally, as with other treatments used for depression, miRNA therapeutics face the complexity of patient-specific responses, as depression’s heterogeneity suggests variability in miRNA profiles among individuals. This necessitates a more personalized approach to therapy. Additionally, the long-term effects of miRNA modulation, in terms of both therapeutic benefits and potential risks, are yet to be fully explored.

## 6. Conclusions

This comprehensive review illuminates the pivotal role of miRNAs in the pathogenesis and treatment of depression, highlighting their potential as biomarkers and therapeutic targets. The intricate regulatory mechanisms of miRNAs in neural development, synaptic plasticity, and stress responses underscore their significance in mental health. The exploration of miRNAs in depression reveals their influence on gene expression and their ability to cross the blood–brain barrier, making them promising candidates for diagnostic and therapeutic purposes. The dysregulation of specific miRNAs in MDD patients suggests a profound connection between miRNA expression patterns and the pathophysiology of depression. For instance, miR-132 and miR-124 have been consistently reported to be altered in MDD, affecting neuroplasticity and stress response mechanisms.

The identification of miRNA signatures specific to MDD opens new avenues for diagnosis. Certain miRNAs, including miR-1202, miR-135a-5p, miR-184, let-7g-5p, miR-103a-3p, miR-107, miR-16-5p, miR-34a-5p, miR-451a, miR-221-3p, and let-7d-3p, have been identified in CSF and serum as dysregulated in depression. This dysregulation suggests a novel approach to diagnosing MDD. Contrasting with conventional diagnostic methods, miRNA profiling offers a quick and non-invasive alternative, potentially employing technologies such as lateral flow assays in the future to measure miRNA concentrations in peripheral blood or serum/plasma. This innovative technique not only aims to improve diagnostic precision but also to enable the early identification of MDD, potentially even before the clinical symptoms manifest.

Moreover, miRNAs hold promise in predicting treatment efficacy. Variations in miRNA expression levels have been associated with patients’ responses to antidepressants, suggesting that miRNA profiling could guide personalized treatment strategies. For example, the differential expression of miR-16 in response to selective serotonin reuptake inhibitors (SSRIs) highlights its potential role in mediating treatment outcomes. Changes in miR-223-3p expression levels at baseline are also predictive of patients’ responses to electroconvulsive therapy. The exploration of miRNAs in MDD underscores a forward-looking approach to diagnosing and managing depression, emphasizing the need for robust research to harness their full diagnostic and therapeutic potential. The challenges in miRNA research, such as inconsistencies between studies, technological limitations, drug delivery complexities, and the need for personalized therapies, emphasize the necessity for further research and standardization in this field. The future of depression treatment may significantly benefit from advancements in miRNA-based strategies, provided these challenges are effectively addressed through continued interdisciplinary collaboration and innovative research.

## Figures and Tables

**Table 1:** List of studies comparing miRNA expression data between depressed and healthy subjects.

Refs.	Sample	Data Source	miRNA (Upregulated)	miRNA (Downregulated)	Gene Target	Effect on Target
[76,77,85]	Dentate gyrus granule cells	Postmortem	hsa-miR-182	-	*BDNF*	BDNF downregulation
[78]	Anterior cingulate cortex	Postmortem	hsa-miR-6077, hsa-miR-4632-3p, hsa-miR-6789-3p, hsa-miR-648, hsa-miR-4498, hsa-miR-6084, hsa-miR-4433a-3p, hsa-miR-4638-5p, hsa-miR-4258, hsa-miR-6850-5p, hsa-miR-3651, hsa-miR-4497, hsa-miR-668-5p, hsa-miR-7108-3p, hsa-miR-4761-3p, hsa-miR-4426, hsa-miR-572, hsa-miR-1470, hsa-miR-520f-3p, hsa-miR-4746-3p, hsa-miR-1193, hsa-miR-6075, hsa-miR-6737-5p, hsa-miR-6879-3p, hsa-miR-4701-3p	hsa-miR-367-3p, hsa-miR-5590-5p, hsa-miR-20b-3p, hsa-miR-548ar-3p, hsa-miR-3689a-5p		
[79]	Prefrontal cortex	Postmortem	-	hsa-miR-1202	*GRM4*	GRM4 upregulation
[80]	Blood	MDD vs. controls	-	hsa-miR-135a	Serotonin transporter	
		MDD 0 weeks vs. MDD 12 weeks	hsa-miR-135a	-		Increased expression of related genes after selective serotonin reuptake inhibitors
[81,86]	Peripheral blood mononuclear cells	MDD vs. controls	hsa-miR-589, hsa-miR-579, hsa-miR-941, hsa-miR-133a, hsa-miR-494, hsa-miR-107, hsa-miR-148a, hsa-miR-652, hsa-miR-425-3p	hsa-miR-517b, hsa-miR-636, hsa-miR-1243, hsa-miR-381, hsa-miR-200c	hsa-miR-425-3p targets MAPK/Wnt signaling pathway	
		MDD 0 weeks vs. MDD 8 weeks	hsa-miR-20b-3p, hsa-miR-433, hsa-miR-409-3p, hsa-miR-410, hsa-miR-485-3p, hsa-miR-133a, hsa-miR-145	hsa-miR-331-5p		
[82]	Blood	MDD 0 weeks vs. MDD 4 weeks (responders vs. non-responders)	-	hsa-miR-223-3p	*IL-6*, *IL-1b*, *NLRP3* and *TNF-α*	Upregulation of these genes in ECT responders at baseline
[83]	Plasma NDEV	MDD vs. controls	-	hsa-miR-93		
[84,87]	Plasma NDEV	MDD 0 weeks vs. MDD 8 weeks (responders vs. non-responders)	hsa-miR-30d-5p, hsa-miR-486-5p	hsa-miR-21-5p	*NR3C1*, *SIRT1*, *SERPINE1*, *RPS6KB1*, *ATF6*, *PSEN1*	

Abbreviations: MDD, major depressive disorder; NDEV, neuron-derived extracellular vesicles; ECT, electroconvulsive therapy.

**Table 2:** Comparative studies on peripheral miRNA expression in depression: pre- and post-antidepressant treatment effects.

Refs.	Tissue Source	miRNAs	Status (Patient vs. Control)	Antidepressant Treatment	Regulation by Treatment	miRNAs
[79,92]	Whole blood	miR-1202	Down	Citalopram	Up	miR-1202
[87,89]	Whole blood	-	-	Escitalopram (12 weeks)	Up	miR-130b, miR-505, miR-29b-2, miR-26b, miR-22, miR-26a, miR-664, miR-494, let-7d, let-7g, let-7e, let-7f, miR-629, miR-106b, miR-103, miR-191, miR-128, miR-502-3p, miR-374b, miR-132, miR-30d, miR-500, miR-589, miR-183, miR-574-3p, miR-140-3p, miR-335, miR-361-5p
					Down	miR-34c-5p, miR-770-5p
[90]	Whole blood	miR-132-3p, miR-124-3p	Up	Citalopram (8 weeks)	Up	miR-124
					Down	miR-132-3p
[94]	Whole blood	-	-	Duloxetine (8 weeks)	Up	miR-3688, miR-5695
[95]	Whole blood	-	-	Escitalopram (2 weeks)	Up	miR-103a-3p, miR-103b, miR-106a-5p, miR-106b-3p, miR-140-3p, miR-145-5p, miR-148b-3p, miR-151a-5p, miR-15a-5p, miR-15b-5p, miR-17-5p, miR-182-5p, miR-185-3p, miR-185-5p, miR-186-5p, miR-20a-5p, miR-20b-5p, miR-210-3p, miR-25-3p, miR-30a-5p, miR-30b-5p, miR-3158-3p, miR-3158-5p, miR-324-5p, miR-331-5p, miR-500a-3p, miR-502-3p, miR-532-5p, miR-550a-3p, miR-584-5p, miR-589-5p, miR-660-5p, miR-93-5p
					Down	miR-1301-3p, miR-191-3p, miR-200b-3p, miR-222-3p, miR-25-5p, miR-27a-3p, miR-30c-1-3p, miR-3168, miR-328-3p, miR-505-5p, miR-744-5p, miR-92a-1-5p
[96]	Plasma	miR-144-5p	Down	Personalized (8 weeks)	Up	miR-144-5p
[97]	Serum	miRNA-34a-5p, miRNA-221-3p	Up	Paroxetine (8 weeks)	Down	miRNA-34a-5p, miRNA-221-3p
		miRNA-451a	Down		Up	miRNA-451a
[84]	Neuron-derived EV	-	-	Escitalopram (8 weeks)	Up (responders)	miR-30d-5p and miR-486-5p

## Data Availability

This study did not report any data.

## Abbreviations

MDD: Major Depressive Disorder; miRNA: microRNA; DSM-V: Diagnostic and Statistical Manual of Mental Disorders, Fifth Edition, 2000; ECT: Electroconvulsive therapy; EVs: Extracellular vesicles; miRISC: miRNA-induced silencing complex; AGO: Argonaute; ESCRT: Endosomal Sorting Complex Required for Transport; LTD: Long-term synaptic depression; RSK3: Ribosomal S6 kinase 3; GRM4: Glutamate metabotropic receptor 4; SSRIs: Selective serotonin reuptake inhibitors; CSF: Cerebrospinal fluid; BDEVs: Brain-derived extracellular vesicles; L1CAM: L1 cell adhesion molecule; NDEVs: Neuron-derived EVs; GLAST: Glutamine aspartate transporter; GLUL: Glutamine synthetase; GFAP: Glial fibrillary acidic protein; ADEVs: Astrocyte-derived EVs; AQP4: Aquaporin 4; LNA: Locked nucleic acid; CUMS: Chronic unpredictable mild stress; AAVs: Adeno-associated viruses; Ads: Adenoviruses; BBB: Blood–brain barrier; RVG: Rabies virus glycoprotein; LFA: Lateral flow assay; GNP: Gold nanoparticle.
